# Cattaneo–Christov Double Diffusion (CCDD) on Sutterby Nanofluid with Irreversibility Analysis and Motile Microbes Due to a RIGA Plate

**DOI:** 10.3390/mi13091497

**Published:** 2022-09-09

**Authors:** Muhammad Faizan Ahmed, A. Zaib, Farhan Ali, Omar T Bafakeeh, Niaz B. Khan, El Sayed Mohamed Tag-ElDin, Mowffaq Oreijah, Kamel Guedri, Ahmed M. Galal

**Affiliations:** 1 Department of Mathematical Sciences, Federal Urdu University of Arts, Sciences & Technology, Gulshan-e-Iqbal, Karachi 75300, Pakistan; 2 Department of Industrial Engineering, Jazan University, Jazan 82822, Saudi Arabia; 3 School of Mechanical & Manufacturing Engineering, National University of Sciences and Technology (NUST), Islamabad 44000, Pakistan; 4 Faculty of Engineering and Technology, Future University in Egypt, New Cairo 11835, Egypt; 5 Mechanical Engineering Department, College of Engineering and Islamic Architecture, Umm Al-Qura University, P.O. Box 5555, Makkah 21955, Saudi Arabia; 6 Research Unity: Materials, Energy and Renewable Energies, Faculty of Science of Gafsa, University of Gafsa, Gafsa 2100, Tunisia; 7 Department of Mechanical Engineering, College of Engineering in Wadi Alddawasir, Prince Sattam bin Abdulaziz University, Al-Kharj 16273, Saudi Arabia; 8 Production Engineering and Mechanical Design Department, Faculty of Engineering, Mansoura University, P.O. Box 35516, Mansoura 35516, Egypt

**Keywords:** Sutterby nanofluid, MHD, riga plate, second law analysis, microorganisms, HAM

## Abstract

In this article, a Riga plate is exhibited with an electric magnetization actuator consisting of permanent magnets and electrodes assembled alternatively. This Riga plate creates an electric and magnetic field, where a transverse Lorentz force is generated that contributes to the flow along the plate. A new study field has been created by Sutterby nanofluid flows down the Riga plate, which is crucial to the creation of several industrial advancements, including thermal nuclear reactors, flow metres, and nuclear reactor design. This article addresses the second law analysis of MHD Sutter by nanofluid over a stretching sheet with the Riga plate. The Cattaneo–Christov Double Diffusion heat and mass flux have been created to examine the behaviour of relaxation time. The bioconvection of motile microorganisms and chemical reactions are taken into consideration. Similarity transformations are used to make the governing equations non-dimensional ordinary differential equations (ODE’s) that are subsequently solved through an efficient and powerful analytic technique, the homotopy analysis method (HAM). The effect of pertained variables on velocity, temperature, concentration, and motile microorganism distributions are elaborated through the plot in detail. Further, the velocity distribution enhances and reduces for greater value Deborah number and Reynold number for the two cases of pseudoplastic and dilatant flow. Microorganism distribution decreases with the augmented magnitude of Peclet number (Pe), Bioconvection Lewis number (Lb), and microorganism concentration difference number (ϖ). The entropy production distribution is increased for the greater estimations of the Reynolds number (ReL) and Brinkman parameter (Br). Two sets of graphical outputs are presented for the Sutterby fluid parameter. Finally, for the justification of these outcomes, tables of comparison are made with various variables.

## 1. Introduction

The rate of heat transport characteristics is a subject of enduring interest for various researchers and scientists owing to its tremendous industrial applications, for example, in mechanical, optical, electrical, and cooling instruments. The rate of heat transport increment is very crucial in depositing energy. Thus, researchers are focused on the investigation of a new fluid substance that is a mixture of nanoparticles 100 nm in size and more thermophysical properties than ordinary fluid, known as nanoliquid. Nanoliquid is the colloidal suspension of the nanoparticles’ thermal behaviour of the ordinary fluid. The first endeavour was performed by Choi et al. [1] in 1995. They exposed the thermal conductivity of nanoliquid by adding nanosized particles. Later, Buongiorno [2] used this notation of nanofluid to make a mathematical form by adding terms known as Brownian and Thermophoretic. The mixed convection nanofluid flows with different surfaces and geometry were conducted by Hussain et al. [3,4], and Haq et al. [5] deliberated the second law analysis on a cross nanofluid. The impact of microorganisms on the Darcy flow of Sutterby nanofluid, taking into account microorganisms over movable cylinders, was examined by Aldabesh et al. [6]. Mankiw et al. [7] analysed the MHD time-dependent flow of nanofluid with variable properties due to an inclined stretching sheet under thermal radiation. Shahid [8] demonstrated the effect of upper convective flow of Maxwell liquid over a permeable surface near the stagnation point. Rafique et al. [9] addressed the stratified micropolar nanofluid flow past an exponentially stretchable plate with the Riga surface. The unsteady viscous flow of nanofluid over the Riga plate using a rotating plate was investigated by Parvine et al. [10]. Abbas et al. [11] deliberated the entropy production over the Riga plate and the suction case. Sannad et al. [12] using a non-homogeneous dynamic model, which is more accurate in physically describing nanofluids than homogeneous ones, wherein free convective flow in a cubical cavity filled with copper-water nanofluid was explored numerically. Some of the latest developments in nanofluids were obtained from references [13,14,15,16,17,18,19].

Different fluid forms, including polymer melts, colloidal suspensions, organic chain mixes, etc., are used in a wide range of industries and production processes. The rheological behaviour of these fluids cannot be well described by the Naiver–Stokes equation alone. Therefore, several nonlinear fluid models are proposed to represent the rheological characteristics of complicated fluids. One non-Newtonian fluid model used to examine key characteristics of pseudoplastic and dilatant fluids is the Sutterby fluid model. Numerous experts have extensively studied the flow of Sutterby liquid. Waqas et al. [20] inspected Sutterby nanofluid using two rotating disks. Yahya et al. [21] investigated Williamson Sutterby nanoparticles under the Cattaneo–Christov heat flux. The effect of MHD on Sutterby nanoparticles due to porous movable sheets was discovered by Fayydh et al. [22]. Gowda et al. [23] examined the Cattaneo–Christof theory of heat diffusion in Sutterby nanofluid. The impact of thermal radiation and heat source/sink on Sutterby nanofluid has been studied by Ali et al. [24]. Hayat et al. [25] investigated the Sutterby fluid with thermal radiation due to a rotating disk. Recently, Fujii et al. [26] addressed Sutterby fluid with natural convection flow due to a vertical plate. The Darcy surface with MHD flow of Sutterby fluid was reported by Bilal et al. [27]. The bioconvection flow of Sutterby nanofluid due to a rotating disk was described by Khan et al. [28]. Sohail et al. [29] designed the free convection flow of Sutterby fluid with heat under the Cattaneo–Christov theory. The heat generation/absorption in the thermally stratified flow of stagnant Sutterby fluid through a linearly stretched plate is analysed in this article by Saif et al. [30]. Usman et al. [31] investigated the two-dimensional stagnant flow of Sutterby nanofluid across a stretching wedge placed in a porous media. Song et al. [32] discussed the two-dimensional flow of Sutterby nanofluids over a stretching cylinder, scrutinized in the presence of bioconvection and swimming microorganisms. The influence of homogeneous heterogeneous reaction on Sutterby fluid flow by a disk with Cattaneo–Christov heat flux is studied by Khan et al. [33].

In the modern period, the research on bio-convection exists due to the motion of microorganisms upwards because microorganisms are denser than water. The upward surface of the fluid develops thickness as a result of the collection of the microorganism. For this reason, the upper surface becomes disturbed, and microorganisms sink, which develops bioconvection. Bio-convection continues to be the subject of research broadly due to its many applications in the clinical area, manufacturing process, and biofuel production. Bio-convection could be organised as consisting of the motion of direction with a vast number of microorganism species. In this way, gyrotactic microorganisms are among those whose swimming direction is based on viscous torque and gravitational force. Kuznetsov et al. [34,35] reported the early investigation of bio-convection in the mixed suspension of nanoparticles with gyrotactic microorganisms. Kotha et al. [36] examined the MHD flow of nanofluid with motile gyrotactic microorganisms over a vertical plate. Siddiq et al. [37] numerically analysed the bioconvection of micropolar nanofluid flow restricted through the stretchable disk using the bvp4c method. Bagh et al. [38] studied the effect of bioconvection and Cattaneo–Christov with a vertical stretching sheet. Azam et al. [39] investigated the effect of bioconvection flow for the Sutterby nanoliquid with nonlinear radiation. Khashi’ie et al. [40] tested a hybrid nanofluid that had bioconvection with gyrotactic microorganisms. Azam [41] explored the time-dependent flow of the chemically reactive Sutterby nanofluid and the influence of gyrotactic microorganisms. Hayat et al. [42] operated the bio-convection flow of nanomaterial subject to melting effect. They addressed thermal nonlinear radiation and Joule heating for heat distribution characteristics. Reddy et al. [43] analysed the time-dependent flow of Cross nanofluid comprising the gyrostatic microorganisms due to slip velocity. Sarkar et al. [44] defined Sutterby nanofluid flow as having motile gyrotactic microorganisms over the Riga plate. Shah et al. [45] described the heat transfer properties of a magnetohydrodynamic Prandtl hybrid nanofluid over a stretched surface in the presence of bioconvection and chemical reaction effects. Ali et al. [46] explored the fluctuating temperature with Cattaneo–Christov features and self-motivated bioconvective microbes immersed in the water-based nanofluid, observed with the excision/accretion of the leading edge.

Riga plate is an electromagnetic actuator, which is made by the combination of a span-wise aligned array of alternating electrodes and permanent magnets. This actuator is responsible for producing a Lorentz force that decreases exponentially. It is important to note that when an external magnetic field is applied to a fluid with high electrical conductivity, it has a provocative effect. However, this device has been used to reduce pressure drag by preventing boundary layer separation and reducing the generation of turbulence requirements. Riga plate was first introduced by Gailitis and Leilausis [47], and they generated a wall paralleled by Lorentz force to control the fluid flow. Further, in contrast to this, the Grinberg term was introduced with a most important feature that the boundary layer of the momentum equation is fully separate from the flow, and exponentially decreases in the direction of the normal to the plate. Subsequently, the Lorentz force was detected over a Riga plate, and then the researchers regained interest in the Gailitis–Lielausis actuator. Islam and Nasrin [48] described the one-dimensional unsteady micropolar fluid flow set in a porous medium along with an inclined infinite Riga plate. Nasrin et al. [49] explored the impulsively started horizontal Riga plate in two-dimensional unsteady Casson fluid flows with rotation. Anjum et al. [50] concentrated on thermal stratification in the flow of second-grade fluid past a Riga plate with linear stretching toward a stagnation region. Hayat et al. [51] investigated the convective heat transfer of electron magnetohydrodynamic squeezed flow past a Riga plate. Wahidunnisa et al. [52] stduied a mixed convective nanofluid flow along a heated Riga plate with viscous dissipation and heat source. Iqbal et al. [53,54] have found the stagnation point flow of the melting heat, thermal radiation, and viscous dissipation effects on the Riga plate using erratic thickness, and considered the Casson fluid stagnation point flow along with a Riga plate. Vishnu et al. [55] discussed the γ Al_2_O_3_–Water/Ethylene Glycol over a Gailitis and Lielausis device with an effective Prandtl number.

Newtonian fluids have been mostly discussed as base fluids in the prior work. We were motivated to examine Sutterby as a base fluid along with the Riga plate and chemical reaction effects with Cattaneo–Christove double diffusion for microorganism flow of nanofluids due to stretching surface because there have been so few studies on them. These facets of the issue, as far as the authors can tell, are not taken into consideration in the investigations that have already been done. The improvement of heat and mass transportation is the main goal of this extensive investigation. The current investigation was encouraged to express the Sutterby nanofluid inserted on a porous surface with Cattaneo–Christof double diffusion due to the Riga plate containing gyrotactic microorganisms. The chemical reaction and heat source-sink are considered worthy of attention. The leading aim of this work is an inclusive analysis of this flow problem. The coupled equations of the current flow problem are altered into the nonlinear system by applying suitable correspondence transformations. Further, the solution of ordinary differential equations is utilized via the Homotopy Analysis Method (HAM). The novel outcomes of the current work are obtained through different parameters and explained with the assistance of graphs and tables.

## 2. Description of the Physical Model

**Figure 1:**
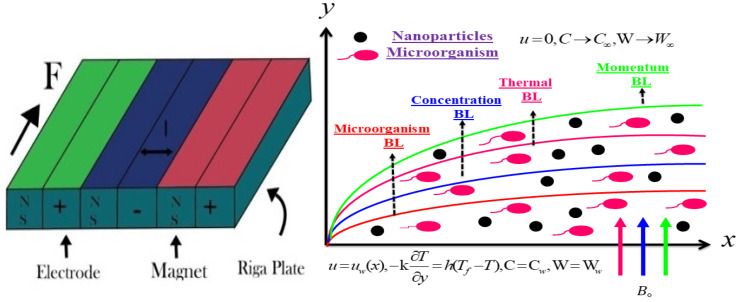
Physical configuration of the flow problem.

Consider the incompressible and steady flow of second law analysis in Sutterby nanofluid due to the Riga Plate containing gyrotactic microorganisms displayed in Figure 1. Cattaneo–Christov with heat and mass flux has also been pondering the temperature and concentration equation. The *x*-axis is considered along with the sheet and the *y*-axis is taken perpendicular to the sheet. Moreover, the sheet of velocity is $U_{w}=ax$. At the surface of the sheet, the temperature of the surface, the concentration of the surface, and the microorganisms of the surface are symbolized by $T_{w}$, $C_{w}$, $\chi_{w}$. The swimming route and velocity of microorganisms do not alter when nanoparticles are present. However, if the volume percentage of nanoparticles exceeds 1%, the motility of microorganisms is impacted. As a result, the base liquid is blended with microorganisms and solid nanoparticles to provide the necessary bioconvection stability. Furthermore, it has been assumed that the fluid contains gyrotactic bacteria. The fluid microorganisms gravitate towards the light. Gyrotactic phenomena, or movement against gravity, are made possible by the “bottom heavy” bulk microorganism, which orients their bodies. The existence of microorganisms is advantageous for the suspension of the nanoparticles. The motion of microorganisms has been taken, irrespective of that of the nanoparticles, to ensure the stability of convection. The flow of a double-diffusive fluid across a stretching sheet containing gyrotactic microorganisms has not yet been investigated, and this study aims to fill that gap with the simplifying of boundary layer approximations of the leading expressions given by [23]:

## 3. Fluid Flow Problem

Cauchy Stress tensor τ for the Sutterby fluid [56,57]

(1) $$\tau =\mu \left(\dot{\gamma }\right)A_{1}-\mathrm{pI},$$

Sutterby viscosity model is represented as

(2) $$\mu =\mu_{\circ }{\left[\left(\frac{\sinh^{-1}\left(\dot{\beta }\dot{\gamma }\right)}{\left(\dot{\beta }\dot{\gamma }\right)}\right)\right]}^{n},$$

where *n*, $\mu_{\circ }$, $\dot{\beta }$, are described as power law index, zero share rate viscosity, and time material constant.

Using Equation (2) in Equation (1), we have

(3) $$\tau =\mu_{\circ }{\left[\left(\frac{\sinh^{-1}\left(\dot{\beta }\dot{\gamma }\right)}{\left(\dot{\beta }\dot{\gamma }\right)}\right)\right]}^{n}A_{1}-p.$$

The governing equation of the following form [23]

(4) $$\frac{\partial u}{\partial x}+\frac{\partial v}{\partial y}=0,$$

(5) $$u\frac{\partial u}{\partial x}+v\frac{\partial u}{\partial y}=\frac{\mu_{0}}{\rho }\left[\frac{\partial^{2}u}{\partial y^{2}}+\frac{mB^{2}}{2}{\left(\frac{\partial u}{\partial y}\right)}^{2}\frac{\partial^{2}u}{\partial y^{2}}\right]+\frac{\pi j_{0}M_{0}}{8\rho }\exp\left(-\frac{\pi }{a}y\right)-\frac{\sigma B_{\circ }^{2}}{\rho }u,$$

(6) $$\begin{array}{l} u\frac{\partial T}{\partial x}+v\frac{\partial T}{\partial y}+\Phi_{E}\Omega_{E}=\frac{k}{\rho C_{p}}\left(\frac{\partial^{2}T}{\partial y^{2}}\right)+\tau \left(D_{B}\frac{\partial C}{\partial y}\frac{\partial T}{\partial y}+\frac{D_{T}}{T_{\infty }}{\left(\frac{\partial T}{\partial y}\right)}^{2}\right)\\ -\frac{1}{\rho C_{p}}\frac{\partial q_{r}}{\partial y}+\frac{Q_{0}}{\rho C_{p}}\left(T-T_{\infty }\right),\; \end{array}$$

(7) $$u\frac{\partial C}{\partial x}+v\frac{\partial C}{\partial y}+\Phi_{C}\Omega_{C}=D_{B}\frac{\partial^{2}c}{\partial y^{2}}+\frac{D_{T}}{T_{\infty }}\left(\frac{\partial^{2}T}{\partial y^{2}}\right)-K_{0}\left(C-C_{\infty }\right),\;$$

(8) $$u\frac{\partial \chi }{\partial x}+v\frac{\partial \chi }{\partial y}+\frac{b\chi_{c}}{\left(C_{w}-C_{\infty }\right)}\frac{\partial }{\partial y}\left(\chi \frac{\partial C}{\partial y}\right)=D_{m}\frac{\partial^{2}\chi }{\partial y^{2}},$$

where *u* and *v* are velocity components in the *x* and *y*- directions. ν represents kinematic viscosity of the fluid, υ represents kinematic viscosity of the fluid, ρ represents the density of the fluid, α represents thermal diffusivity, C is the concentration, $D_{B},D_{t}$ represent Brownian diffusion and thermophoretic diffusion, $C_{p}$ denotes volumetric expansion, $D_{m}$ represents the microorganism coefficient, $\Omega_{E}$ and $\Omega_{C}$ fluid relaxation time,

In the above equations, the terms $\Omega_{E}$ $\Omega_{C}$ are stated as

(9) $$\Omega_{E}=u\frac{\partial u}{\partial x}\frac{\partial T}{\partial x}+v\frac{\partial u}{\partial y}\frac{\partial T}{\partial x}+u\frac{\partial v}{\partial x}\frac{\partial T}{\partial y}+v\frac{\partial v}{\partial y}\frac{\partial T}{\partial y}+u^{2}\frac{\partial^{2}T}{\partial x^{2}}+2uv\frac{\partial^{2}T}{\partial x\partial y}+v^{2}\frac{\partial^{2}T}{\partial y^{2}},$$

(10) $$\begin{array}{l} \Omega_{C}=u\frac{\partial u}{\partial x}\frac{\partial C}{\partial x}+v\frac{\partial u}{\partial y}\frac{\partial C}{\partial x}+u\frac{\partial v}{\partial x}\frac{\partial C}{\partial y}\\ +v\frac{\partial v}{\partial y}\frac{\partial C}{\partial y}+u^{2}\frac{\partial^{2}C}{\partial x^{2}}+2uv\frac{\partial^{2}C}{\partial x\partial y}+v^{2}\frac{\partial^{2}C}{\partial y^{2}} \end{array},$$

The relevant boundary conditions are assumed to be the form

(11) $$\begin{array}{l} u=U_{w}(x),\;v=0,-k\frac{\partial T}{\partial y}=h\left(T_{w}-T_{\infty }\right),\;C=C_{w},\;W=W_{w}\;\mathrm{as}\;y=0,\\ u\to 0,\;T\to T_{\infty },\;C\to C_{\infty },\;W\to W_{\infty }\;at\;y\to \infty . \end{array}\}$$

(12) $$q_{r}=\frac{4\sigma_{1}}{3k*}\frac{\partial T^{4}}{\partial y}=-\frac{16\sigma^{*}}{3k^{*}}T^{3}\frac{\partial T^{4}}{\partial y},$$

where $T^{4}$ can be expanded as follows:

(13) $$T^{4}\;≅4T^{3}{}_{\infty }T-3T^{4}{}_{\infty }.$$

Replacing Equation (12) into Equation (13),

(14) $$q_{r}=\frac{16\sigma *T^{3}{}_{\infty }}{3k*}\frac{\partial T}{\partial y}.$$

Introducing the similarity variables [24]

(15) $$\begin{array}{l} u=axf^{\prime }(\eta ),\;v=-\sqrt{av}f(\eta ),\;\eta =y\sqrt{\frac{a}{v}},\\ \theta =\frac{T-T_{\infty }}{T_{w}-T_{\infty }},\;\phi =\frac{C-C_{\infty }}{C_{w}-C_{\infty }},\;W=\frac{\chi -\chi_{\infty }}{\chi_{w}-\chi_{\infty }}, \end{array}\}$$

Using Equation (15), Equations (4)–(6) become

(16) f‴+f f″−f′2+12βδReLf″2f‴+Ze−Aη−Mf′=0,

(17) $$\theta^{\prime \prime }\left(1+\frac{4}{3}Rd\right)+\mathrm{Pr}Nt{\theta^{\prime }}^{2}+\mathrm{Pr}Nb\theta^{\prime }\phi^{\prime }-\mathrm{Pr}\lambda_{1}\left(ff^{\prime }\theta^{\prime }+f^{2}\theta^{\prime \prime }\right)+\mathrm{Pr}ϒ\theta =0,$$

(18) $$\phi^{\prime \prime }+Scf\phi^{\prime }+\left(\frac{Nt}{Nb}\right)\theta^{\prime \prime }-\mathrm{Pr}Sc\lambda_{2}\left(ff^{\prime }\phi^{\prime }+f^{2}\phi^{\prime \prime }\right)-ScCr\phi =0,$$

(19) $$W^{\prime \prime }-Pe\left[\phi^{\prime \prime }\left(W+\varpi \right)+\phi^{\prime }W^{\prime }\right]-LbfW^{\prime }=0,$$

The corresponding boundary conditions are

(20) $$\begin{array}{l} f(0)=0,f^{\prime }(0)=1,\theta^{\prime }(0)=Bi(\theta (0))-Bi,\phi (0)=1\\ f^{\prime }(\infty )=0,\theta (\infty )=0,\phi (\infty )=0,W(\infty )=0. \end{array}\}\;$$

where $Z=\frac{\pi J_{\circ }M_{\circ }h}{8\rho U_{\circ }^{2}}$ is the Modified Hartmann number; ReL=ax2v is the Reynolds number; $\delta =\frac{B^{2}a^{2}}{v}$ is the Deborah number; $Rd=\frac{4\sigma^{*}T_{\infty }^{3}}{kk^{*}}$ is the Radiation parameter; $\mathrm{Pr}=\frac{v}{\alpha }$ is the Prandtl number; $Nb=\frac{\tau D_{B}(C_{w}-C_{\infty })}{v}$ is the Brownian motion parameter; $Nt=\frac{\tau D_{t}(T_{w}-T_{\infty })}{T_{\infty }v}$ is the Thermophoresis parameter; $Sc=\frac{v}{D_{B}}$ is the Schmidt number; $Bi=\frac{h_{f}}{k}\sqrt{\frac{v}{a}}$ is the Biot number; $Pe=\frac{bW_{c}}{D_{m}}$ is the Peclet number; $Lb=\frac{v}{Dm}$ is the Bio convection Lewis number; $Cr=\frac{K_{\circ }}{c}$ is the Chemical reaction; $M=\frac{\sigma B_{\circ }^{2}}{\rho a}$ is the Magnetic number.

The thermo-fluidic quantities of engineering interest in this study are skin friction $Cf_{x}$, heat transfer rate $Nu_{x}$, mass transfer rate $Sh_{x}$, and motile density $Wh_{x}$.

(21) $$\begin{array}{l} C_{fx}=\frac{\tau_{w}}{pu_{w}^{2}},Nu_{x}=\frac{xq_{w}}{k\left(T_{w}-T_{\infty }\right)},Sh_{x}=\frac{xq_{m}}{D_{b}\left(C_{w}-C_{\infty }\right)}\;\mathrm{and}\;Wh_{x}=\frac{xq_{n}}{D_{m}\left(X_{w}-X_{\infty }\right)}.\} \end{array}$$

where $\tau_{w}$ surface shear stress, $q_{w}$ surface heat flux, $q_{m}$ surface mass flux, and $q_{n}$ motile density are given by the following expressions:

(22) τw=−μ0[∂u∂y+16βδReL(∂u∂y)3]y=0,qw=−k(∂T∂y)|y=0,qm=−k(∂C∂y)|y=0and qn=−Dm(∂χ∂y)|y=0.}

The dimensionless form of the above parameters is expressed as

(23) CfRex0.5=[f″(0)+16βδReL(f″(0))3],NuxRex1/2=−[1+43Rd]θ′(0),ShxRex1/2=−ϕ′(0) and WnRex1/2=−W′(0).}

Here, Rex=xUwv is the local Reynolds number.

## 4. Entropy Generation Analysis

Entropy generation with Sutterby fluid is communicated as [39]:

(24) S‴gen=kT∘([∂T∂x]2+[∂T∂y]2+16σ*T∞33kk*(∂T∂y)2)+μT∞(∂u∂y)2[1+βδReL6(∂u∂y)2]+RDmC∞(∂C∂y)2+RDmT∞(∂T∂y)(∂C∂y)+RDmχ∞(∂χ∂y)2+RDmT∞(∂χ∂y)(∂T∂y)+σB∘2T∞u2.

From Equation (23) the entropy production consists of some terms.

The first term represents heat transfer, the second-term fluid friction, and the third to sixth terms represent diffusive irreversibility. The significance of entropy production can be written as

(25) $${S^{\prime \prime \prime }}_{\circ }=\frac{\kappa {\left(\Delta T\right)}^{2}}{L^{2}T_{\infty }^{2}}.$$

Using Equation (15) the rate of entropy production with Sutter by Equation (23) can be converted

(26) NG=S‴genS‴∘=ReL(1+Rd)θ′2+ReLBrΠ[1+βδReL3(f″)2]+ReL(ΓΠ)2ϕ′2+ReL(ΓΠ)ϕ′θ′+ReL(ξΠ)2W′2+ReL(ξΠ)W′θ′+BrΠMf′2.

The Bejan number Be is defined as the ratio over the entropy generation with heat transport $S_{T}$ and the total entropy production $N_{G}$, and it can be written as:

(27) $$Be=\frac{S_{T}}{N_{G}}$$

(28) Be=ReL(1+Rd)θ′2+ReL(ΓΠ)2ϕ′2+ReL(ΓΠ)ϕ′θ′+ReL(ξΠ)2W′2+ReL(ξΠ)W′θ′ReL(1+Rd)θ′2+ReLBrΠ[1+βδReL3(f″)2]+ReL(ΓΠ)2ϕ′2+ReL(ΓΠ)ϕ′θ′+ReL(ξΠ)2W′2+ReL(ξΠ)W′θ′+BrΠMf′2

## 5. Homotopy Expression

HAM was used to investigate the explicit and analytic solutions of Equations (16)–(19) [51]. This analytical technique is quite effective and flexible in solving the parabolic-type boundary value problems of any order, is unconditionally stable, and attains remarkable accuracy. Taking the initial guesses and the linear operators as

(29) $$f_{\circ }(\eta )=\left(1-e^{-\eta }\right),\theta_{\circ }(\eta )=\left[\frac{Bi}{1+Bi}\right]e^{-\eta },\phi_{\circ }(\eta )=e^{-\eta },W_{\circ }(\eta )=e^{-\eta },$$

(30) $$\begin{array}{l} {\hat{L}}_{f}=f^{\prime \prime \prime }-f^{\prime },\;{\hat{L}}_{\theta }=\theta^{\prime \prime }-\theta ,\;\\ {\hat{L}}_{\phi }=\phi^{\prime \prime }-\phi ,\;{\hat{L}}_{W}=W^{\prime \prime }-W, \end{array}$$

with the property

(31) $$\begin{array}{l} {\hat{L}}_{f}\left(R_{1}+R_{2}e^{-\eta }+R_{3}e^{-\eta }\right)=0,\\ {\hat{L}}_{\theta }\left(R_{4}+R_{5}e^{-\eta }\right)=0,\\ {\hat{L}}_{\phi }\left(R_{6}+R_{7}e^{-\eta }\right)=0,\\ {\hat{L}}_{W}\left(R_{8}+R_{9}e^{-\eta }\right)=0, \end{array}\}$$

in which $B_{i},\left(i=1-9\right)$ are the constants.

## 6. The Zeroth Order Formulation

(32) $$\begin{array}{l} (1-s){\hat{L}}_{f}\left[f(\eta ;s)-f_{\circ }(\eta )\right]=s\hbar_{f}{\;\underline{N}}_{f}\left[f(\eta ;s),\theta (\eta ,s),\phi (\eta ,s),W(\eta ,s)\right],\\ (1-s){\hat{L}}_{\theta }\left[\theta (\eta ;s)-\theta_{\circ }(\eta )\right]=s\hbar_{\theta }{\;\underline{N}}_{\theta }\left[f(\eta ;s),\theta (\eta ,s),\phi (\eta ,s)\right],\\ (1-s){\hat{L}}_{\phi }\left[\phi (\eta ;s)-\phi_{\circ }(\eta )\right]=s\hbar \phi {\;\underline{N}}_{\phi }\left[f(\eta ;s),\theta (\eta ,s),\phi (\eta ,s)\right],\\ (1-s){\hat{L}}_{W}\left[W(\eta ;s)-W_{\circ }(\eta )\right]=s\hbar_{W}{\;\underline{N}}_{W}\left[f(\eta ;s),\theta (\eta ,s),\phi (\eta ,s),W(\eta ,s)\right]. \end{array}\}$$

(33) $$\begin{array}{l} f(0,s)=0,\;f^{\prime }(0,s)=1,\;f^{\prime }(\infty ,s)=0,\\ \theta^{\prime }(0,s)=Bi\left[\theta (0,s)-1\right],\;\theta (\infty ,s)=0,\\ \phi (0,s)=1,\;\phi (\infty ,s)=0,\\ W(0,s)=0,\;W(\infty ,s)=0, \end{array}\}$$

here ${\underline{N}}_{f}$, ${\underline{N}}_{\theta }$, ${\underline{N}}_{\phi }$, and ${\underline{N}}_{W}$ are defined below

(34) N¯f[f(η,s)]=∂3f(η,s)∂η3+f(η,s)∂2f(η,s)∂η2−[∂f(η,s)∂η]2+12βδReL[∂f(η,s)∂η]2[∂3f(η,s)∂η3]+Ze−Aη−Mf′

(35) $$\begin{array}{l} {\underline{N}}_{\theta }\left[f(\eta ,s),\theta (\eta ,s)\right]=\frac{\partial^{2}\theta (\eta ,s)}{\partial \eta^{2}}\left(1+\frac{4}{3}Rd\right)+\mathrm{Pr}Nt{\left[\frac{\partial \theta (\eta ,s)}{\partial \eta }\right]}^{2}\\ +\mathrm{Pr}Nb\left[\frac{\partial \theta (\eta ,s)}{\partial \eta }\right]\left[\frac{\partial \phi (\eta ,s)}{\partial \eta }\right]-\mathrm{Pr}\lambda_{1}\left(\begin{array}{l} f(\eta ,s)\left[\frac{\partial f(\eta ,s)}{\partial \eta }\right]\left[\frac{\partial \theta (\eta ,s)}{\partial \eta }\right]\\ +{\left[f(\eta ,s)\right]}^{2}\frac{\partial^{2}\theta (\eta ,s)}{\partial \eta^{2}} \end{array}\right)\\ +\mathrm{Pr}ϒ\theta (\eta ,s), \end{array}$$

(36) $$\begin{array}{l} {\underline{N}}_{\phi }\left[f(\eta ,s),\theta (\eta ,s),\phi (\eta ,s)\right]=\frac{\partial^{2}\phi (\eta ,s)}{\partial \eta^{2}}+Scf(\eta ,s)\left[\frac{\partial \phi (\eta ,s)}{\partial \eta }\right]\\ +\left(\frac{Nt}{Nb}\right)\left[\frac{\partial^{2}\theta (\eta ,s)}{\partial \eta^{2}}\right]-\mathrm{Pr}Sc\lambda_{2}\left(\begin{array}{l} f(\eta ,s)\frac{\partial f(\eta ,s)}{\partial \eta }\frac{\partial \phi (\eta ,s)}{\partial \eta }\\ +f^{2}(\eta ,s)\frac{\partial^{2}\phi (\eta ,s)}{\partial \eta^{2}} \end{array}\right)-ScCr\phi (\eta ,s), \end{array}$$

(37) $$\begin{array}{l} {\underline{N}}_{W}\left[f(\eta ,s),\phi (\eta ,s),W(\eta ,s),\right]=\frac{\partial^{2}W(\eta ,s)}{\partial \eta^{2}}-Pe\left[\begin{array}{l} \frac{\partial^{2}\phi (\eta ,s)}{\partial \eta^{2}}\left(W+\varpi \right)\\ +\frac{\partial \phi (\eta ,s)}{\partial \eta }\frac{\partial W(\eta ,s)}{\partial \eta } \end{array}\right]-\\ Lbf(\eta ,s)\frac{\partial W(\eta ,s)}{\partial \eta }. \end{array}$$

For s=0 and s=1, the results are achieved

(38) $$\begin{array}{l} f(\eta ;0)=f_{0}(\eta ),\;\theta (\eta ;0)=\theta_{0}(\eta ),\;\phi (\eta ;0)=\phi_{0}(\eta ),\;W(\eta ;0)=W_{0}(\eta )\\ f(\eta ;1)=f(\eta ),\;\theta (\eta ;1)=\theta (\eta ),\;\phi (\eta ;1)=\phi (\eta ),\;W(\eta ;1)=W(\eta ) \end{array}\}$$

## 7. The *m*th Order Formulation

The mth order deformation can be presented in the following forms

(39) $$\begin{array}{l} {\hat{L}}_{f}\left[f_{m}\left(\eta ,s\right)-\chi_{m}f_{m-1}(\eta )\right]=\hbar_{f}R_{f,m}(\eta ),\\ {\hat{L}}_{\theta }\left[\theta_{m}\left(\eta ,s\right)-\chi_{m}\theta_{m-1}(\eta )\right]=\hbar_{\theta }R_{\theta ,m}(\eta ),\\ {\hat{L}}_{\phi }\left[\phi_{m}\left(\eta ,s\right)-\chi_{m}\phi_{m-1}(\eta )\right]=\hbar_{\phi }R_{\phi ,m}(\eta ),\\ {\hat{L}}_{W}\left[\theta_{m}\left(\eta ,s\right)-\chi_{m}W_{m-1}(\eta )\right]=\hbar_{W}R_{W,m}(\eta ). \end{array}\}$$

Boundary conditions are

(40) $$\begin{array}{l} {f^{\prime }}_{m}(0)=f_{m}(0)={f^{\prime }}_{m}(\infty )={\theta^{\prime }}_{m}(0)-Bi\theta_{m}(0)=\theta_{m}(\infty )=0,\\ \phi_{m}(0)=\phi_{m}(\infty )=W(0)=W(\infty )=0. \end{array}\}$$

where

(41) Rf,m(η)=f‴m−1(η)+∑k=0m−1fm−1−kf″k−∑k=0m−1f′m−1−kf′k+12βδReγ∑k=0m−1f″m−1−kf″kf‴k+Ze−Aη−M∑k=0m−1f′m−1−k

(42) $$\begin{array}{l} R_{\theta ,m}(\eta )={\theta^{\prime \prime }}_{m-1}(\eta )\left(1+\frac{4}{3}Rd\right)+\mathrm{Pr}Nt\sum_{k=0}^{m-1}{\theta^{\prime }}_{m-1-k}{\theta^{\prime }}_{k}+\mathrm{Pr}Nb\sum_{k=0}^{m-1}{\phi^{\prime }}_{m-1-k}{\theta^{\prime }}_{k}\\ -\mathrm{Pr}\lambda_{1}\left(\sum_{k=0}^{m-1}\left(\sum_{r=0}^{k}f_{m-1-k}{f^{\prime }}_{k-r}\right){\theta^{\prime }}_{k}+\sum_{k=0}^{m-1}{f^{\prime }}_{m-1-k}{f^{\prime }}_{k}{\theta^{\prime \prime }}_{k}\right)+\mathrm{Pr}ϒ\theta_{m-1} \end{array}$$

(43) $$\begin{array}{l} R_{\phi ,m}(\eta )={\phi^{\prime \prime }}_{m-1}(\eta )+Sc\sum_{k=0}^{m-1}{f^{\prime }}_{m-1-k}\phi_{k}+\left(\frac{Nt}{Nb}\right){\theta^{\prime \prime }}_{m-1}\\ -\mathrm{Pr}Sc\lambda_{2}\left(\sum_{k=0}^{m-1}\left(\sum_{r=0}^{k}f_{m-1-k}{f^{\prime }}_{k-r}\right){\phi^{\prime }}_{k}+\sum_{k=0}^{m-1}{f^{\prime }}_{m-1-k}{f^{\prime }}_{k}{\phi^{\prime \prime }}_{k}\right)-ScCr\phi_{m-1} \end{array}$$

(44) $$\begin{array}{l} R_{W,m}(\eta )={W^{\prime \prime }}_{m-1}(\eta )-Pe\left(\sum_{k=0}^{m-1}{\phi^{\prime \prime }}_{m-1-k}W_{k}+{\phi^{\prime \prime }}_{m-1}\varpi +\sum_{k=0}^{m-1}{\phi^{\prime }}_{m-1-k}{W^{\prime }}_{k}\right)\\ -Lb\sum_{k=0}^{m-1}{W^{\prime }}_{m-1-k}f_{k} \end{array}$$

(45) $$\eta_{m}=\left\{\begin{array}{l} 0,m\leq 1\\ 1,m>1 \end{array}\right\}.$$

The general solutions are

(46) $$\begin{array}{l} f_{m}=f_{m}^{*}+R_{1}+R_{2}e^{\eta }+R_{3}e^{-\eta }\\ \theta_{m}=\theta_{m}^{*}+R_{4}e^{\eta }+R_{5}e^{-\eta }\\ \phi_{m}=\phi_{m}^{*}+R_{6}e^{\eta }+R_{7}e^{-\eta }\\ W_{m}=W_{m}^{*}+R_{8}e^{\eta }+R_{9}e^{-\eta } \end{array}\}$$

in which $f_{m}^{*},\;\theta_{m}^{*},\;\phi_{m}^{*},\;W_{m}^{*}$ are the special solution.

(47) $$\begin{array}{l} f_{m}=f_{m}^{*}+R_{1}+R_{2}e^{\eta }+R_{3}e^{-\eta }\\ \theta_{m}=\theta_{m}^{*}+R_{4}e^{\eta }+R_{5}e^{-\eta }\\ \phi_{m}=\phi_{m}^{*}+R_{6}e^{\eta }+R_{7}e^{-\eta }\\ W_{m}=W_{m}^{*}+R_{8}e^{\eta }+R_{9}e^{-\eta } \end{array}\}$$

## 8. Convergence of Homotopy Solutions

**Figure 2:**
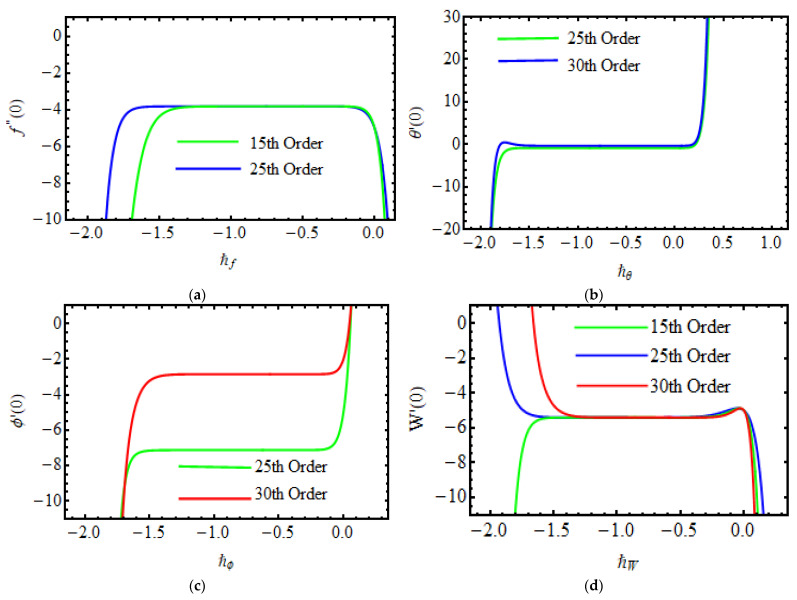
Plots of (**a**) $\hbar_{f}$ the curve of $f^{\prime \prime }(0)$ (**b**) $\hbar_{\theta }$ the curve of θ′(0) (**c**) $\hbar_{\phi }$ the curve of ϕ′(0) (**d**) $\hbar_{w}$ the curve of W′(0).

**Figure 3:**
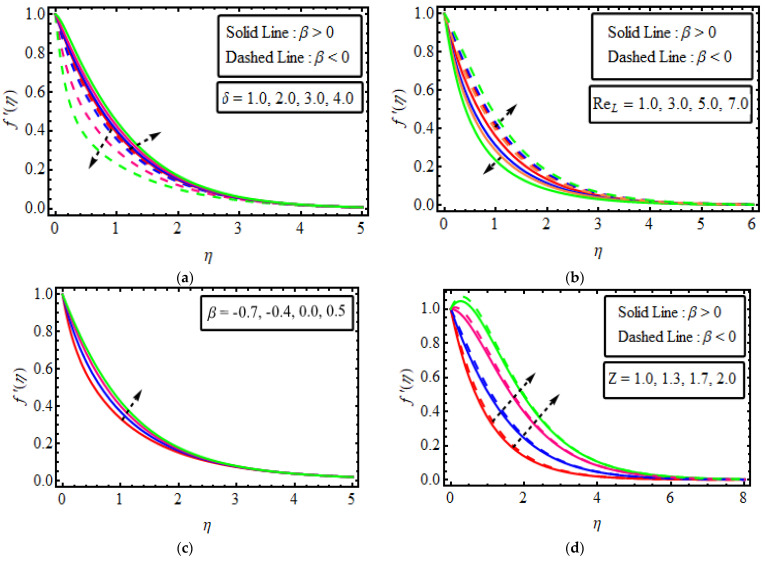
(**a**–**d**). The impact of $f^{\prime }(\eta )$ numerous variables (**a**) δ (**b**) ReL (**c**) β (**d**) Z.

**Figure 4:**
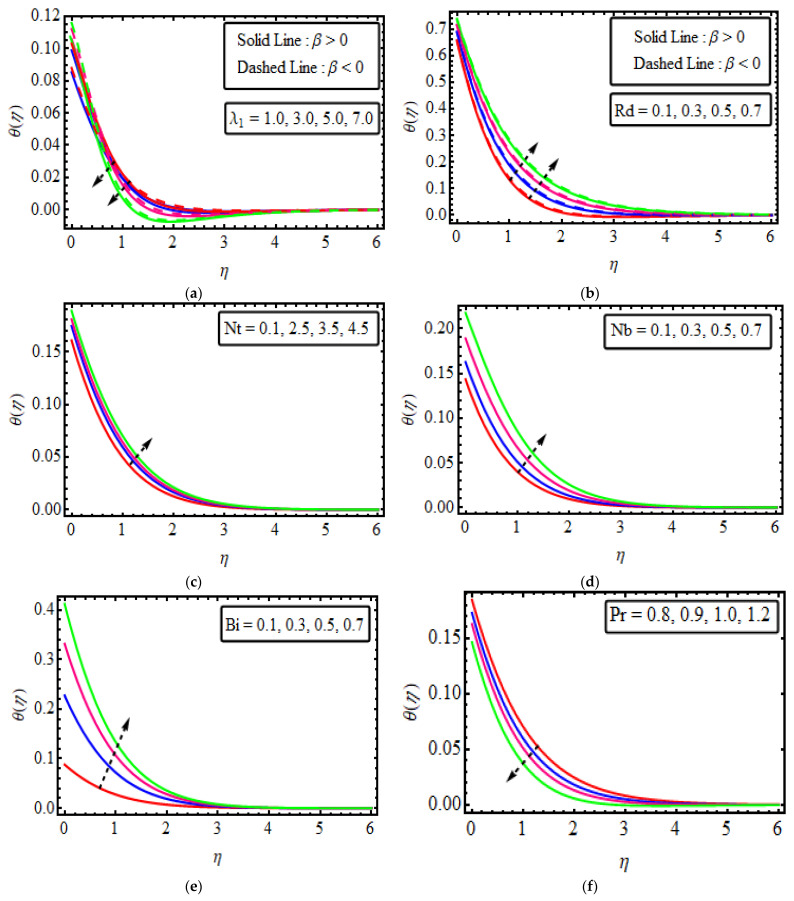
(**a**–**f**). The impact of θ(η) numerous variables (**a**) $\lambda_{1}$ (**b**) Rd (**c**) Nt (**d**) Nb (**e**)  Bi (**f**) Pr.

These parameters $\hbar_{f},\hbar_{\theta },\hbar_{\phi }\;\mathrm{and}\;\hbar_{W}$ are the converging control of the desired series solution. For the function $f^{\prime \prime }(0),\;\theta^{\prime }(0),\;\phi^{\prime }(0),\;W^{\prime }(0)$ to seek the permissible values to obtain the 25th and 30th order. Figure 1, Figure 2, Figure 3 and Figure 4 specify that the range of $\hbar_{f},\hbar_{\theta },\hbar_{\phi }\;\mathrm{and}\;\hbar_{W}$$-2.0<\hbar_{f}<-0.1,-2.0<\hbar_{\theta }<-1.0,-1.7<\hbar_{\phi }<-1.0,-2.0<\hbar_{W}<-1.0.$ The series converges in the entire region of η when $\hbar_{f}=-0.65,\;\hbar_{\theta }=\hbar_{\phi }=-0.55,\;\hbar_{W}=-0.7$. Table 1 indicates the convergence solution of HAM versus different order of approximations.

## 9. Results and Discussion

**Figure 5:**
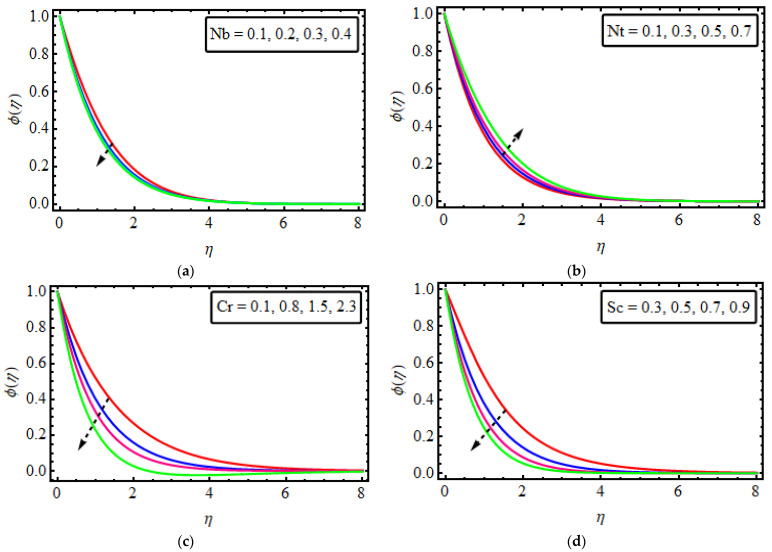
(**a**–**d**) The impact of ϕ(η) numerous variables (**a**) Nb (**b**) Nb (**c**) Cr (**d**) Sc.

**Figure 6:**
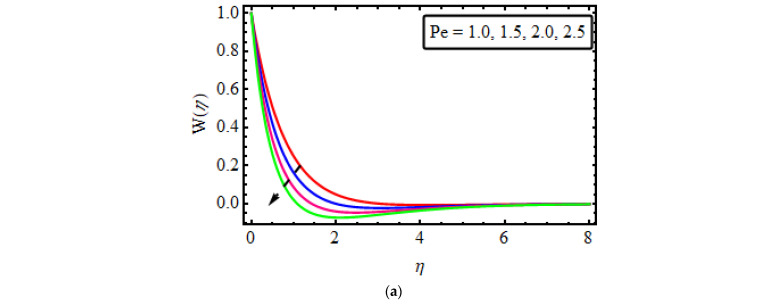

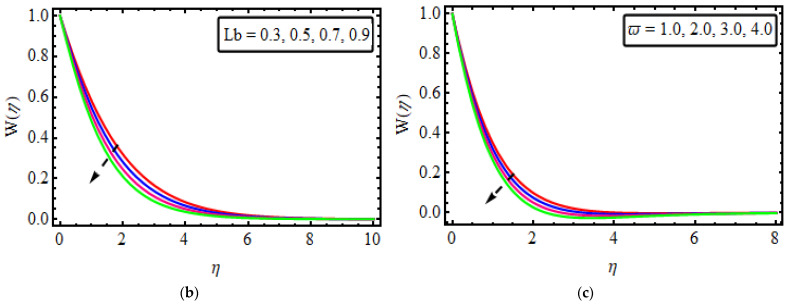
(**a**–**c**) The impact of ϕ(η) numerous variables (**a**) Nb (**b**) Nb (**c**) Cr (**d**) *Sc*.

**Figure 7:**
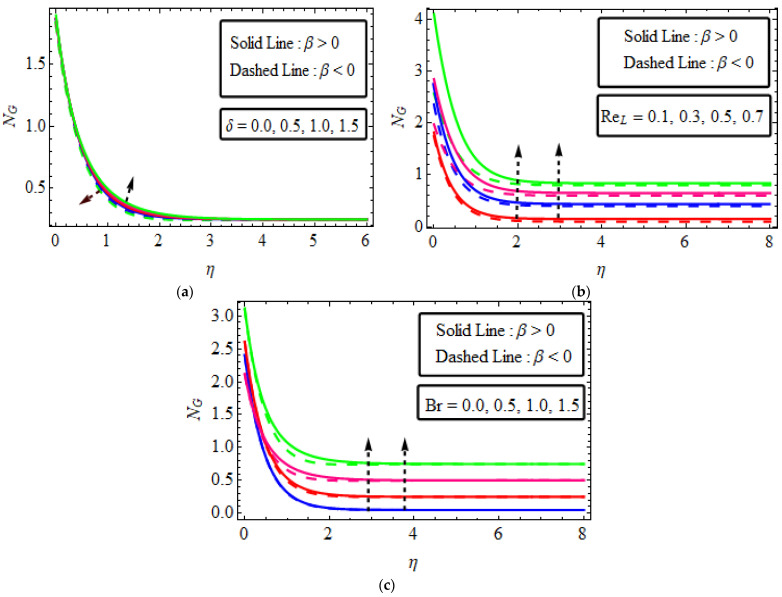
(**a**–**c**) The impact of $N_{G}$ numerous variables (**a**) δ (**b**) ReL (**c**) Br.

**Figure 8:**
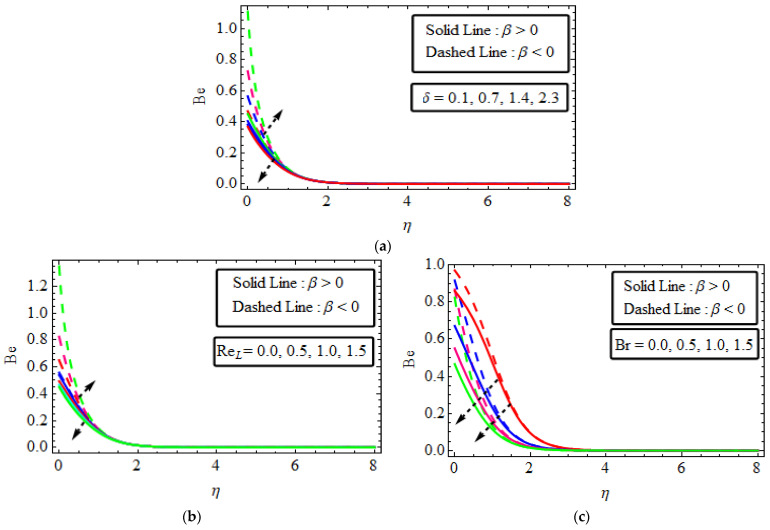
(**a**–**c**) The impact of Be numerous variables (**a**) δ (**b**) ReL (**c**) Br.

**Figure 9:**
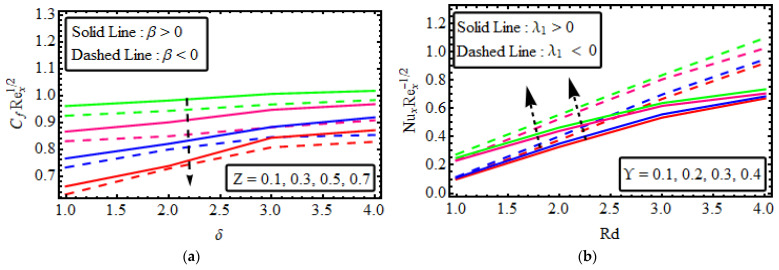

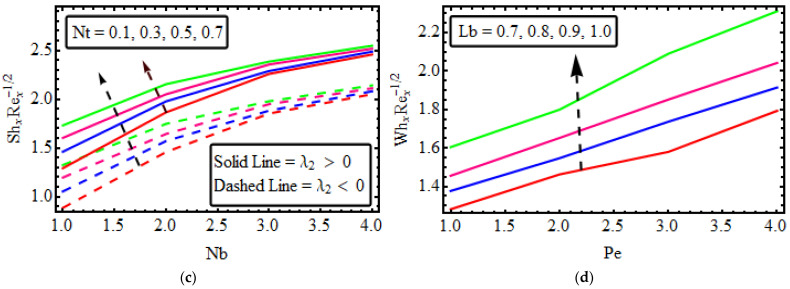
(**a**–**d**) Variation in Rex1/2Cf, Rex−1/2, NuRex−1/2Sh, Rex−1/2Whx numerous variables (**a**)Z, δ (**b**) Nb, ϒ (**c**) Nb, Nt (**d**) Lb, Pe.

The system of Equations (16)–(19) subject to the boundary conditions (20) has been tackled through the analytical technique known as the Homotopy analysis method (HAM) for distinct parameters that emerged in the analytical simulation. To discuss the performance of physical significance against the velocity field $f^{\prime }(\eta )$, temperature distribution θ(η), concentration field ϕ(η), motile microorganism profile W(η), entropy production, Bejan number, as well as skin friction, Nusselt number, Sherwood number and motile density microorganism, are delineated in Figure 3, Figure 4, Figure 5, Figure 6, Figure 7, Figure 8 and Figure 9. Table 2 verifies −θ(0) with Ali and Zaib [55], and found a good agreement.

## 10. Results and Discussion

The system of Equations (16)–(19) subject to the boundary conditions (20) has been tackled through the analytical technique known as the Homotopy analysis method (HAM) for distinct parameters that emerged in the analytical simulation. To discuss the performance of physical significance against the velocity field $f^{\prime }(\eta )$, temperature distribution θ(η), concentration field ϕ(η), motile microorganism profile W(η), entropy production, Bejan number, as well as skin friction, Nusselt number, Sherwood number and motile density microorganism, are displayed graphically and in tabular form. Table 2 verifies −θ(0) with Ali and Zaib [58], and found a good agreement.

## 11. Velocity Field

The effects of different numerous parameters on velocity distribution $f^{\prime }(\eta )$ are discussed in Figure 3a–d. Figure 3a shows that for pseudoplastic, the velocity of fluid lessens with the larger value of Deborah number δ, and the velocity field is enhanced for the rising value of Deborah number δ for the two cases of when β<0 and β>0. This result is shown to be the same as in F. Ali et al. [56]. Figure 3b shows that the velocity curve $f^{\prime }(\eta )$ shortens with increasing values of the Reynold number ReL in the case of β<0; a higher ReL, the more diminished the viscous force and hence fluid depreciates for the pseudoplastic fluid. Thus, for shear thickening fluid, the velocity field enhances as ReL increasing the impact of the values of β in the two cases. As shown in the figure, the velocity for both values β shows mixed behaviour with increasing fluid parameters. The Reynold number described the proportion of inertial to viscous forces due to this inertial force being prominent for the two cases of velocity magnitude when (β=2.5) and increases when (β=−2.5). Figure 3c manifests the effect of the enhanced power-law index β parameter, resulting in a better velocity profile for shear thickening fluid. Figure 3d demonstrates the significance of the Hartmann number Z on velocity fluid field for the two cases of β<0 and β>0. It has been revealed that with changes in the strength of Z the velocity of the fluid escalates in both cases. Physically, an increment in Z corresponds to enhancing the external electric field that constructs wall-parallel Lorentz force. Therefore $f^{\prime }(\eta )$ rises. The velocity is more pronounced for pseudo-plastic fluid than a fluid dilatant fluid with increasing Z for the shear-thinning as well as shear-thickening fluid.

## 12. Temperature Field

Figure 4a–f plotted the consequences of temperature θ(η) against different values of various involved parameters. The thermal relaxation time parameter causes are demonstrated in Figure 4a. The thermal relaxation parameter tends to decrease the temperature curve in both cases of dilatants and pseudoplastic. It impacts the strength of heat transfer. So, the dissimilar decayed impact of $\lambda_{1}$ on the temperature field takes place away from the surface. Figure 4b reveals the inclination of θ(η) for specific values of thermal radiation parameters fo β>0 and β<0 The temperature of the fluid increases due to the enlargement in the radiation parameter Rd in both cases. Physically, by increasing the radiation parameter more energy is added to the system, which uplifts the kinetic energy of molecules and also causes a rise in the temperature profile. Figure 4c shows that temperature increases with the increments in the values of Nt. Thermophoresis is a phenomenon where small particles diffuse under the effect of a temperature gradient. An increase in the value of the thermophoresis parameter sets the nanoparticle in motion, and thus on the hotter side, the momentum of the nanoparticles rises. Due to the rise in the momentum, nanoparticles transfer their kinetic energy in the direction of the cooler side, and thus the region gets warmed up quickly; i.e., the temperature of the fluid is increased. Figure 4d also suggests that the nanofluid temperature rises with the increasing value of the Brownian motion parameter Nb, and the opposite effect is examined in the Figure 4e. The reason behind this is that Brownian motion is the irregular movement of the particles suspended in the fluid. The temperature of the fluid increases as a result of the random collision of particles suspended in the liquid, which further leads to an expected improvement in the temperature profile θ(η)Figure 4e depicts the impact of the Biot number θ(η). These figures point out that the temperature field is boosted by enhancing the value of the Biot number. It seems that the value of the Biot number is directly proportional to the temperature, as the higher value of Bi corresponds to greater convective heating along the sheet, which increases the temperature gradient on the sheet. Therefore, the thickness of the boundary layer and temperature are increasing functions of the Biot number. Figure 4f display variations of the Prandtl number against θ(η). Temperature is restrained against higher Pr. Basically, the Prandtl number refers to thermal diffusivity. Larger Pr indicates lower thermal diffusivity, which causes the temperature to decompose.

## 13. Concentration Field

The outcomes of different leading parameters ϕ(η) are presented in Figure 5a–d. Figure 5a is designed to show the characteristics of Nb on ϕ(η). The concentration distribution is depleted by rising Nb. The reason behind the decrease in the concentration field is the relation of Brownian motion with the Brownian diffusion coefficient which is responsible for decreasing the concentration field. The influence of the thermophoresis variable ϕ(η) is rendered in Figure 5b. It can be seen that increasing values of the thermophoresis parameter enhances mass concentration. This is because the increase in Nt corresponds to the increase in thermophoretic diffusion coefficient enhancing mass concentration. The influence of chemical reaction on the profile of concentration is shown in Figure 5c. The enhanced values Cr result in a fluid particle breakthrough near the surface, which reduces the concentration and the corresponding boundary layer thickness. The further rate of inclination is slower in the presence of the chemical reaction rate parameter. The Schmidt number outcomes for concentration are displayed in Figure 5d. A depreciation in concentration arises with greater Sc due to the reduction of mass diffusion. In the physical phenomenon of sight, an increase in the Sc lessens the molecular diffusivity and results in the declination of the concentration gradient.

## 14. Microorganism Field

The effect of different influential variables on the microorganism’s field is shown in Figure 6a–c. Variations in motile microorganisms against the Lewis number Lb for various values are seen in Figure 6a. The Lb reduces the density of microorganisms. Actually, Lb have an opposite trend with thermal diffusivity, as an escalation in Lb decreases the thermal diffusivity with regard to a decline in motile density. Figure 6b points out the behaviour of the Peclet number in the microorganism field. The motile density profile decreases with an increase in Pe. Actually, the Peclet number is the quotient of heat transfer by fluid motion to the heat transfer through thermal conduction, and the microorganism’s diffusivity is in an inverse relationship with the Peclet number. That is why the motile density field is on the decline for large estimates of Pe The relationship between the microorganisms’ difference parameter ϖ and the motile density is present in Figure 6c. It has been noticed that motile density shrinks for larger ϖ. In fact, improving the values ϖ escalates the concentration of microorganisms in the ambient concentration, and so W(η) declines.

## 15. Entropy Generation

Figure 7a–c examined the performance of numerous variable parameters δReL and Br entropy production $N_{G}$. Figure 7a sketched the effect of Deborah’s number δ versus the entropy generation number $N_{G}$. This shows an enhancement in entropy production close to the wall for dilatant β<0, and deduction close to the wall for pseudo-plastic fluid β<0. The increasing influence of the Reynolds number Reγ in entropy generation studied for dilatant and pseudoplastic fluids is plotted in Figure 7b. The disparity Br has been plotted in Figure 7c. Entropy generation is boosted with increasing values of Br in both cases. Subsequently, Br attributes a proportion of the free heat through viscous heating to the molecular condition. Therefore, heat is created in the system for increased values of Br while the disorderliness of *Re* also rises in the system, which increases the entropy of the system.

## 16. Bejan Number

The performance Be with the variations in the variables δ, Reγ and Br, Be plotted in Figure 8a–c. Figure 8a,b signify the behaviour of the physical parameters Deborah’s number and the Reynold number on the Bejan number. It is shown that the Bejan number declines as the larger values of Deborah’s and the Reynold number for shear-thickening and upsurge in both numbers for shear-thinning fluid. Furthermore, Figure 8c shows that the influence of the Bejan number Be is reduced β>0 and β<0 with the growing values of Br. Actually, the thermal conductivity of the fluid declines with the rise in Br, so that a greater amount of heat is transmitted through the fluid. The parameter Br characterizes the heat generated by viscous dissipation. So as a result, the Bejan number is reduced via the uplift value of the Brickman number Br.

## 17. Physical Entitles

**Figure 10:**
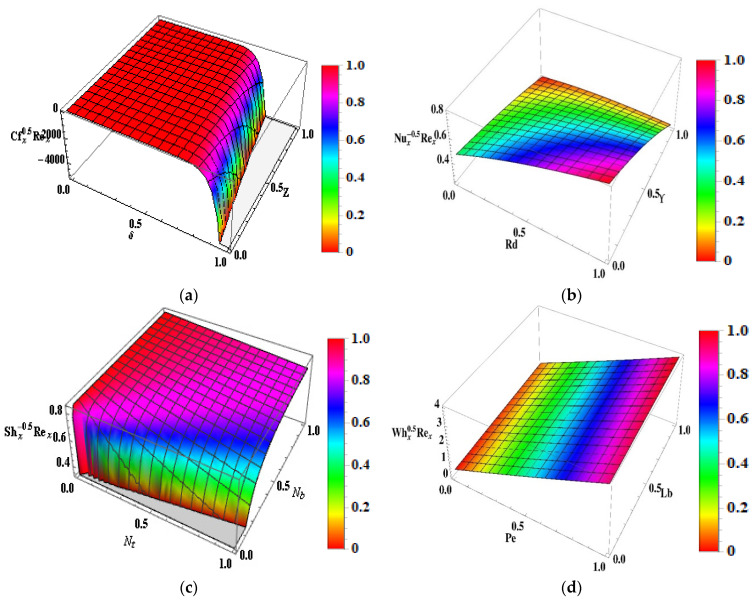
(**a**–**d**) 3D graph (**a**) Skin friction for δ and Z (**b**) Nusselt number for Rd and ϒ. (**c**) Sherwood number for Nt and Nb (**d**) Motile density for Pe and Lb.

Figure 9a shows that the skin friction coefficient Rex1/2Cfx is deformed in both cases for the larger values of the parameter when β>0 and β<0. The influence of the non-Newtonian nanofluid parameter on the Nusselt Rex−1/2Nu against thermal radiation Rd are highlighted in Figure 9b. The heat transport gradient upsurges with rises in the thermal radiation Rd and heat source/sink parameters ϒ. The significance of Nt and Nb on the Sherwood number is intimated in Figure 9c. It is determined that there is an amplification in the Sherwood number Rex−1/2 ⁡Sh for raised values of fluid parameters. Figure 9d elucidates the substantial rescaled density number of motile microorganisms. The rescaled density number of motile microorganisms is voluminous for higher variations of Pe and Lb. Figure 10a–d shows the 3D representation of the Skin, Nusselt, Sherwood and Motile density respectively.

The numerical estimation of drag friction Z, δ, ReL, A has been given in Table 3. The larger the magnitude of Z, δ, ReL, A, the lower the drag friction. Table 4 and Table 5 produce the data of heat and mass related to the diverse value of Rd, Bi, Pr, Cr, Sc. Here it is illustrated that the magnitude of heat and mass transport is increased via a large value of ϒ for the Nusselt number Nt and for the Sherwood number. Table 6 shows the variation of Pe, Lb, ϖ on the motile density profile. In Table 6, it is shown the motile density profile is the leading function Lb.

## 18. Streamline and Isotherm Line

**Figure 11:**
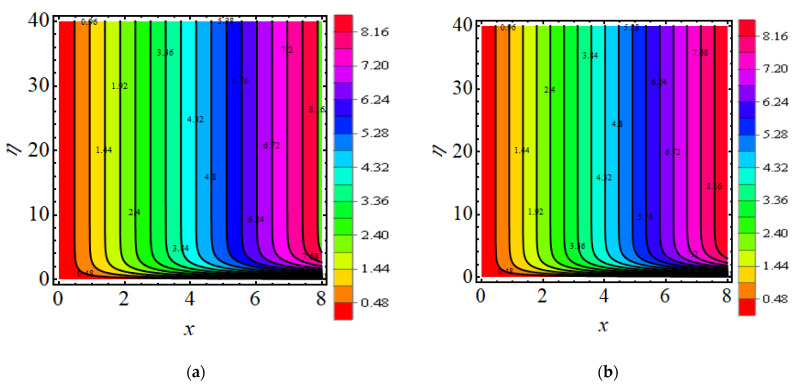
(**a**,**b**) Streamline for (**a**) β=−2.5 (**b**) β=2.5.

**Figure 12:**
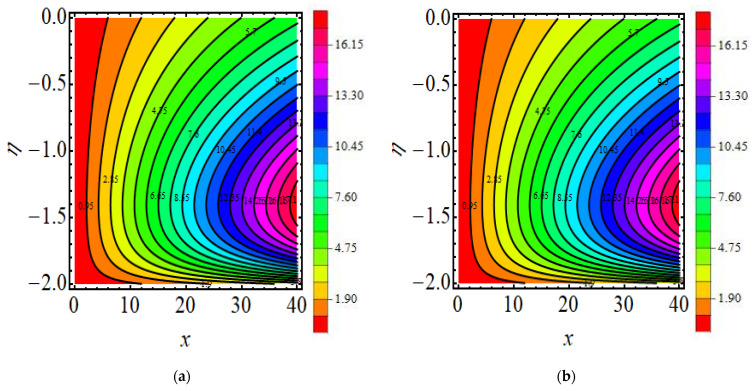
(**a**,**b**) Isotherms lines (**a**) β=−2.5 (**b**) β=−2.5.18. Major Outcomes.

Figure 11a,b exhibits the behaviour of the stream function for the current flow. The patterns show that the streamlines are more obscured and split into two sections pseudo-plastic β<0 and dilatant β>0; the shape is modest and fills the floe field. Figure 12a,b show the behaviour of the isotherm line for present flow for both cases.

This investigation examines MHD Sutterby nanofluid with Cattaneo–Christov Double Diffusion (CCDD) dual diffusion theory due to the Riga Plate, as well as the bioconvection of motile microorganisms; a chemical reaction is also analysed. Appropriately modified transformations are invoked to get a non-linear system of differential problems. The model under consideration consists of both gyrotactic microorganisms and nanoparticles. By using bio-convective flow, which is produced by the combined impacts of nanoparticles and buoyancy forces, microorganisms maintain the suspension of nanoparticles. Refs. [59,60,61,62,63] are extended in future with different flow assumptions. Along with Newtonian heating, the mechanics of Brownian motion and thermophoresis are also considered. The main findings of the current study are the following:

- The convergence of HAM solutions is ensured up to the 25th iteration.
- Deterioration and elevation behaviour in the momentum of fluid is manifested in relation to Deborah’s number and Reynold’s number when β=−2.5 and β=2.5.
- The velocity shows continuous improvement with increases in the Hartman number in cases of dilatant and pseudoplastic fluid.
- The growing estimate of the variable leads to increases in the dimensionless temperature field.
- Declining aptitude expressed in the concentration field against the Schmidt number and Prandtl number.
- A larger chemical reaction Cr portrays a decline in the concentration, while the Biot number Bi lead to the expansion in concentration.
- The microorganism field has deteriorated for the higher value of Pe and microorganism difference parameter.
- The entropy generation number presented an increasing magnitude for large values of the Reynolds number and Brinkman number, for the case of pseudoplastic and dilatants fluid. Large values of the entropy generation number appear in the vicinity of the sheet due to high viscous effects.
- Enhancing the value of Deborah’s number and Reynold’s number results in Bejan’s profile decay in the case of dilatant fluid, while the opposite effect is observed in the case of shear-thinning.
- Skin fraction decelerated for the modified Hartman number Z but accelerated against the Nusselt number for the heat source/sink parameter ϒ and the Sherwood number for the Thermophoresis parameter Nt.
- The density of motile microorganisms goes up with rising values of Lb,Pe  and ϖ.

## Figures and Tables

**Table 1:** Convergence solutions of HAM for different order of approximations when β=0.2,δ=0.1,M=0.3,Pr=3.0,Nb=Nt=0.1,Sc=1.0,ϒ=0.1,Pe=0.3,Lb=0.2.

Order of HAM				
**Approximation**	$-f^{\prime \prime }(0)\;$	$-\theta^{\prime }(0)$	$-\phi^{\prime }(0)$	$-W^{\prime }(0)$
1	0.874702	0.166821	1.21667	0.955833
5	0.768677	0.167973	1.41759	1.038657
10	0.765186	0.168223	1.42596	1.07814
15	0.765357	0.168144	1.42606	1.08236
20	0.765347	0.168158	1.42609	1.08264
25	0.765341	0.168158	1.42608	1.08261
30	0.765341	0.168157	1.42608	1.08258
35	0.765341	0.168157	1.42608	1.08258
40	0.765341	0.168157	1.42608	1.08258

**Table 2:** Numerical outcomes of −θ(0) When Pr = Sc = 10, Bi = 0.1.

		Ali and Zaib [58]	Bvp4c	Current Result (HAM)
Nt	Nb			
0.1	0.1	0.092906	0.0923	0.093757137
0.5	0.5	0.092126	0.0925	0.093975770

**Table 3:** Numerical outcomes Rex1/2Cfx for numerous values of Z,δ,ReL,A.

Z	δ	ReL	A	Rex1/2Cfx	
				β=−2.5	β=2.5
0.1	0.4	0.3	0.2	0.92454	0.96057
0.3				0.82986	0.86558
0.5				0.73214	0.79555
0.7	0.4		0.8	0.63177	0.74173
0.1	0.6	0.3		0.94253	0.98097
0.3				0.84824	0.89991
0.5				0.79896	0.82019
0.7	0.6			0.72767	0.77578
0.1	0.8	0.3	1.6	0.96645	1.00572
0.3				0.88142	0.94353
0.5				0.84492	0.88265
0.7	0.8			0.80704	0.82265
0.1	1.0	0.3	2.4	0.98264	1.01726
0.3	1.0			0.90723	0.96739
0.5				0.85289	0.91848
0.7	1.0			0.82750	0.87050

**Table 4:** Numerical outcomes of Rex−1/2Nux numerous values of Rd,ϒ,Bi,Pr,Nb,Nt.

Rd	ϒ	Bi	Pr	Nb	Nt	Rex−1/2Nux	
						$\lambda_{1}=-2.5$	$\lambda_{1}=2.5$
0.1	0.1	0.1	1.0	0.1	0.1	0.10375	0.09868
0.2			1.3		0.2	0.11564	0.10996
0.3	0.1	0.2	1.6		0.3	0.23375	0.23171
0.4			1.9	0.1	0.4	0.27543	0.25036
0.5	0.2	0.3	2.0	0.2	0.1	0.37615	0.32808
0.6			2.2		0.2	0.40300	0.35108
0.7		0.4	2.6	0.2	0.3	0.52608	0.44059
0.8			2.8		0.4	0.55644	0.46506
0.9	0.3	0.5	3.0	0.3	0.1	0.66801	0.53560
1.0			3.1		0.2	0.69913	0.55870
1.1		0.6	3.4	0.3	0.3	0.80631	0.61842
1.2			4.0	0.3	0.4	0.83636	0.63832
1.3	0.4	0.7	4.2	0.4	0.1	0.92220	0.67117
1.4			4.5		0.2	0.94881	0.68554
1.5		0.8	4.9		0.3	1.02733	0.70633
1.8			5.5	0.4	0.4	1.09963	0.73584

**Table 5:** Numerical outcomes f Rex−1/2Shx for numerous values of Cr,Pr,Sc.

Cr	Pr	Sc	Nb	Nt	Rex−1/2Shx	
					$\lambda_{2}=-2.5$	$\lambda_{2}=2.5$
0.3	2.5	1.0	0.1	0.1	0.88698	1.29324
0.6	2.7	1.1		0.2	1.05551	1.46176
0.9	2.9	1.2		0.3	1.19741	1.60365
1.2	3.0	1.3		0.4	1.32527	1.73152
1.5	3.3		0.2	0.1	1.45829	1.86454
1.8	3.5			0.2	1.57097	1.97722
2.0	3.7	1.4		0.3	1.64331	2.04955
2.3	4.0			0.4	1.74712	2.15337
2.6	4.1	1.8	0.3	0.1	1.85226	2.25851
2.7				0.2	1.88221	2.28846
2.9				0.3	1.94866	2.35491
3.0	4.2	1.9		0.4	1.97869	2.38494
3.2		1.9	0.4	0.1	2.05108	2.45733
3.3	4.3		0.4	0.2	2.08112	2.48737
3.4				0.3	2.11116	2.51741
3.5	4.5	1.9		0.4	2.14119	2.54744

**Table 6:** Numerical outcomes Rex−1/2Whx for numerous values of Pe,Lb,ϖ.

Pe	Lb	ϖ	Nb	Nt	Rex−1/2Whx
1.0	0.7	0.3	0.1	0.1	1.28167
1.2	0.8	0.5		0.2	1.37667
		0.7		0.3	1.45567
	0.9	0.9		0.4	1.60451
1.3		0.3	0.2	0.1	1.46258
		0.5		0.2	1.54708
1.4	0.9	0.7		0.3	1.65217
	1.0	0.9		0.4	1.79951
1.5		0.3	0.3	0.1	1.57958
1.6		0.5		0.2	1.73667
1.7		0.7		0.3	1.85154
1.8		0.9		0.4	2.08983
	1.5	0.3	0.4	0.1	1.79321
		0.5		0.2	1.91432
1.9	1.6	0.7		0.3	2.04217
2.0	1.7	0.9		0.4	2.31083

## Data Availability

All the data available inside the research work.

## Abbreviations

a	Constant
u,v	Velocity components
f	Similarity function for velocity
$T_{\infty }$	Ambient temperature
$T_{w}$	Wall constant temperature
T	Fluid Temperature
χ	Microorganism concentration
θ	Dimensionless temperature
ϕ	Dimensionless concentration
$C_{\infty }$	Ambient concentration
C	Fluid concentration
β	Power index number
$B_{\circ }$	$\mathrm{Magnetic}\;\mathrm{field}\;\mathrm{strength}\;\left({\mathrm{NA}}^{-1}m^{-1}\right)$
δ	Deborah number
ReL	Local Reynolds number
Z	Modified Hartmann number
M	Magnetic Parameter
Rd	Thermal Radiation
ϒ	Heat source/sink parameter
Cr	Chemical reaction
$\lambda_{1}$	Thermal Relaxation parameter
$\lambda_{2}$	Concentration Relaxation parameter
Nt	Thermophoresis parameter
Nb	Brownian motion parameter
Bi	Biot number
Pr	Prandtl number
Sc	Schmidt number
Pe	Peclet number
Lb	Lewis number
ϖ	Microorganism concentration difference parameter
$D_{B}$	Mass diffusivity
$D_{T}$	Thermophoresis diffusivity
$J_{\circ }$	Current density
$C_{f}$	Skin friction coefficient
$Nu_{x}$	Nusselt number
$Sh_{x}$	Sherwood number
$Wh_{x}$	Microorganism density number
k	Thermal conductivity of the fluid $\left(m^{2}/s\right)$
Br	Brickman number
$S_{G}$	Local volumetric entropy generation rate
$N_{G}$	Entropy number
Be	Bejan number
τ	Ratio of the effective heat capacity
η	Similarity variable
ρ	Fluid Density
μ	Dynamic viscosity
∞	Ambient condition
